# Dr. William H. Daly

**Published:** 1901-07

**Authors:** 


### OBITUARY NOTES.

Dr. William H. Daly, of Pittsburg, committed suicide by shooting himself in the right temple at his residence, June 9, 1901, aged 59 years. Dr. Daly was one of the most distinguished laryngologists in the country and had been president of the American Laryngological Association, as well as of several other medical organisations. He served during the civil war as a medical cadet, and during the Spanish war as chief surgeon of volunteers attached to the staff of General Miles. Dr. Daly was a man of splendid physique and of cheerful demeanor; but it is thought by many that the loss of his wife, a year and a half ago, served to depress him. His death was a great shock to his many friends all over the country.

